# Correction: A novel, efficient and magnetically recyclable Cu–Ni bimetallic nanocomposite as a highly active bifunctional catalyst for Pd-free Sonogashira and C–N cross-coupling reactions: a combined theoretical and experimental study

**DOI:** 10.1039/d3ra90077k

**Published:** 2023-09-01

**Authors:** Mohammad Ali Nasseri, Mansoore Shahabi, Seyyedeh Ameneh Alavi G., Ali Allahresani

**Affiliations:** a Department of Chemistry, Faculty of Sciences, University of Birjand P. O. Box 97175-615 Birjand Iran manaseri@birjand.ac.ir

## Abstract

Correction for ‘A novel, efficient and magnetically recyclable Cu–Ni bimetallic nanocomposite as a highly active bifunctional catalyst for Pd-free Sonogashira and C–N cross-coupling reactions: a combined theoretical and experimental study’ by Mohammad Ali Nasseri *et al.*, *RSC Adv.*, 2023, **13**, 22158–22171, https://doi.org/10.1039/D3RA01965A.

The authors regret that the title shown in the original article was incorrect. The correct title is as shown above.

An independent expert has viewed the corrected title and has concluded that it is consistent with the discussions and conclusions presented.

The Royal Society of Chemistry apologises for these errors and any consequent inconvenience to authors and readers.

## Supplementary Material

